# Investigating the Relationship Between Sexual Function and Quality of Life in Menopausal Women 

**Published:** 2016-12

**Authors:** Masumeh Ghazanfarpour, Talat Khadivzadeh, Masaudeh Babakhanian

**Affiliations:** 1 Student Research Committee, Department of Midwifery and Reproductive Health, Nursing and Midwifery School, Mashhad University of Medical Sciences, Mashhad, Iran; 2 Department of Midwifery, School of Nursing and Midwifery, Mashhad University of Medical Sciences, Mashhad, Iran; 3 Social Determinant of Health Research Center, Semnan University 0f Medical Sciences, Semnan, Iran

**Keywords:** Sexual Function, Quality of Life, Menopausal Women

## Abstract

**Objective:** To evaluate the symptoms of menopausal women and the link between sexual function, menopausal symptoms and demographic variables.

**Materials and methods:** This is a cross-sectional study in which 202 postmenopausal women admitted to the health care centers were selected. The Female Sexual Function Index (FSFI) questionnaire and Menopause-Specific Quality of Life (MENQOL) were the main means of data gathering.

**Results:** The results of our study suggested that suggested that women experienced a number of menopausal symptoms such hot flash, headache and neck pains, reduced physical strength weight gain, pain or leg cramps, intensified sexual problem than women who lack such symptoms. The FSFI scores were lower in women who were more than 60 years old, had low educational level (illiterate and elementary), and smoked cigarette. The most common symptoms were hot flashes (45%), Sleeplessness (37%), and pain in joints and muscles (36%). Moreover, the highest mean score belonged to symptoms associated with hot flashes (1.49 ± 1.38), sleeplessness (1.48 ± 1.71), and headache and neck pains (1.14 ± 1.59) table 2.

**Conclusion:** Women with a history of sexual problem experienced more intense menopausal symptoms. This study sheds more light on the link between sexual problems and menopausal symptoms, which can helps healthcare professionals to offer a desirable package to their patients.

## Introduction

The World Health Organization (WHO) recognizes sexuality as a dynamic and integrated part of human life (1). Female sexual dysfunction (FSD) is a term used to refer to series of disorders associated with sexual desire, orgasm, arousal, and sexual pain, which can brings about considerable personal distress (2). Sexual dysfunction is escalated with age and illness, but medication and surgery are more likely to affect sexual functioning in comparison to ageing alone (3). The risk factors associated with sexual dysfunction during menopausal years include increasing age and decreasing estrogen. The dimensions of sexual function negatively affected by menopause are orgasm, lubrication and sexual pain (4). The incidence of sexual dysfunction among postmenopausal women is about 50% in the United States (US), with approximately 9.7 million women aged 50 to 74 suffering from its consequences (5). Numerous studies have reported the link between general quality of life, menopausal symptoms, and sexual function (2, 6-8). The relationship between menopause symptoms and sexual dysfunction in menopausal women have been the subject of many studies in western countries (9-11). Mishra et al reported a relationship between somatic symptoms, hot flushes and sexual problems (12). According to Hess et al, women with vaginal dryness reported lower levels of sexual enjoyment (13) Changes in the shape of body, skin appearance and fitness may damage self –esteem and confidence, and therefore inhibit the initiation of sexual activities (14). Despite of the increased life expectancy and the growth of elderly population in Iran, there is a paucity of studies on sexuality of older people (15). Nonetheless, there is still a paucity of studies about menopause symptoms and sexual dysfunction in Iranian context. This study is an attempt to examine the relationship between women’s sexual function, menopausal symptom and demographic variables.

## Materials and methods

This study draws on a cross-sectional method to investigate the relationship between sexual dysfunction, menopausal symptoms and demographic variable. This study was ratified by the Ethics Committee of Semnan University of Medical Science. (ethical code: ajums.1393. 497421). For this purpose, 202 randomly-selected postmenopausal women attending health care centers in the city of Semnan were selected. Post-menopause period in women is characterized by intact uterus and amenorrhea for more than 12 consecutive months. The main inclusion criterion was natural menopause status and women with a history of hysterectomy and HRT (current users) were excluded from the study. The questionnaire was made of two parts with the first part containing items about demographic variables like age, educational level, smoking habit (smoker or non-smoker), and occupation status of women and their husbands. Furthermore, the first part included the Female Sexual Function Index (FSFI) proposed by Rosen et al. The FSFI is a 19-item scale developed for the specific purpose of assessing domains of sexual functioning such as sexual desire, arousal, lubrication, orgasm, satisfaction, and pain during in clinical trials (16). higher scores on the questionnaire and subscales indicate better sexual function .In several studies, the Persian version of FSFI questionnaire has been validated (17). The total FSFI scores and their subdomains had acceptable level of internal consistency (from 0.93 to 0.95). Fakhri et al assessed the overall test–retest reliability coefficients and demonstrated high reliability of each IV-FSFI domain (ranging from 0.73 to 0.86) (18). The second part contained Menopause-Specific Quality of Life (MENQOL) questionnaire. Designed by Hilditch et al in 2008, it consisted of 29 items which were divided into four categories of vasomotor (3 items), psychosocial (7 items), physical (16 items) and sexual (3 items) sections. It requires women to recall their experiences in the past month. If their answer to an item is negative, they will proceed to the next item; otherwise, they are asked to rate the severity of their symptoms on a 7-point Likert scale ranging from zero (barely) to six (extremely) (19). In this questionnaire, higher scores are associated with lower quality of life (20). The Persian version of MENQOL questionnaire has been assessed and validated in many studies (21-23).

The statistical analysis was carried out by SPSS 11 software (SPSS Inc., Chicago, IL). The data analysis was performed by student’s t-test and ANOVA. The Shapiro Wilktest was used to determine the normality of data distribution, the total FSFI scores and its subdomains. A value of p < 0.05 was assumed to be statistically significant.

## Results

The post-menopausal women had an average age of 52.69 ± 37 years. Six women were excluded from the study as they were HRT user. Among subjects, 13.21% were illiterate and 49% were nonsmokers, with only 20.1% of them having more than two children (Table 1). 

Nonsmokers scored higher than smokers in FSFI index (Cigar- Hookah) (p < 0.017). Also, women with low educational levels (illiterate and primary school) had lower FSFI

Scores and thus greater sexual problems than women with a middle school, high school and university degree table 1. Moreover, FSFI scores were higher in women aged more than 60 years old. In the same vein, menopausal women whose husbands were unemployed reported significantly lower FSFI scores than women whose had a job (p = 0.05). 

**Table 1:** The relationship between sexual symptoms and demographic and health characteristics

**Variables**	**%**	**Sexual score**	**Test**
Age group (years)			
40-50	52.7	50.19 ± 16.37	Df = 159 T = 3.38 P = 0.001
50-70	47.3	40.38 ± 19.7	
Women level of education			
Illiterate	20.61	35.66 ± 19.43	Df = 4 F = 5.96 P < 0.0001
Primary school	25.77	42.39 ± 22.16	
Middle school	24.22	49.95 ± 15.76	
High school	20.61	53.25 ± 9.27	
University	8.76	52.14 ± 15.08	
Smoker			
nonsmoker	74.5	47.73 ± 17.77	Df = 170 T=2.41 P=0.017
Smoker (Cigar- Hookah)	25.5	40.13 ± 18.25	
No. of Children			
More than 2	32.44	48.41 ± 16.67	Df = 1 F = 1.31 P = 0.25
Less than 2	67.55	44.93 ± 18.82	
Women Occupation			
Retired	7.93	44.92 ± 19.04	Df = 3 F = 1.12 P = 0.34
Employed	14.81	50.50 ± 9.21	
Part-time job	3.70	54.60 ± 26.46	
Unemployed	73.54	44.63 ± 18.77	
Retired	41.29	42.41 ± 20.12	Df = 3 F = 4.05 P = 0.08
Employed	31.84	51.92 ± 12.09	
Part-time job	19.40	46.11 ± 17.71	
Unemployed	7.46	37.50 ± 23.19	
Income			
< 300$	20	45.20 ± 17.53	Df = 4 F = 0.73 P = 0.57
300-600$	57.03	50.34 ± 16.53	
600-800$	8.88	44.25 ± 19.74	
800-1000$	9.62	51.08 ± 7.70	
> 1000$	4.44	48 ± 14.79	

There was no significant association between menopausal women’s sexual function and their employment, income level and number of children. The findings of the present study suggested that women experienced a number of menopausal symptoms such hot flash (p = 0.01), headache and neck pains (p = 0.03), reduced physical strength (p = 0.02), weight gain (p = 0.01), pain or leg cramps (p = 0.03), intensified sexual problems compared to women without such symptoms (data were not shown).As shown by the results, the most common symptoms were hot flashes (45%), Sleeplessness (37%), and pain in joints and muscles (36%). Moreover, the highest mean score belonged to symptoms associated with hot flashes (1.49 ± 1.38), sleeplessness (1.48 ± 1.71), and headache and neck pains (1.14 ± 1.59) table 2.

## Discussion

This study aimed to assess the relationship between menopause symptoms, sexual function and demographic variables of women in the city of Semnan. With the prolonged expectancy, more women suffer from the long-run consequences of menopause. The results of 2011 Census in Iran indicate an upsurge in the elderly population (24). 

Menopause is often accompanied with a series of symptoms such as hot flashes, night sweat, vaginal atrophy, tension, apprehension and lessened libido (25). The study of Ghazanfarpour suggested that menopause symptoms varied in terms of frequency and severity across Iran (26). In our study, hot flashes were the most common climacteric symptoms, which is consistent with the literature (2, 27). 

**Table 2:** Frequency and severity of menopausal symptoms

**Symptom**	**n (%)**	**Mean ± SD** **(minimum– maximum)**
Hot flashes	46	1.49 ± 1.38
Night sweats	20	0.78 ± 1.17
Sweating	25	0.93 ± 1. 21
Dissatisfaction with personal life	16	1.18 ± 1.23
Anxiety and nervousness	29	1.14 ± 1.44
loss of memory	32	1.06 ± 1.26
Less effective than before	27	1.07 ± 1.44
Feelings of depression	27	1.03 ± 1.36
Being impatient with other people	23	0.95 ± 1.34
loneliness	18	0.84 ± 1.36
Feeling of isolation	24	0.95 ± 1.37
Joint and muscle pain	36	1.62 ± 1.95
Feel like crying and worries	22	0.85 ± 1.27
Sleeplessness	37	1.48 ± 1.71
headache and neck pains	31	1.22 ± 1.67
Reduced physical strength	28	1.14 ± 1.59
Decrease in stamina	27	1.22 ± 1.66
Lack of energy	30	1.17 ± 1.49
Dry skin	21	0.86 ± 1.22
Weight gain	30	1.04 ± 1.29
Increased facial hair	25	0.93 ± 1.29
Changes in skin appearance and texture	11	0.61 ± 1.16
Feeling bloated	13	0.51 ± 0.98
Feeling lumbago	35	1.34 ± 1.60
Frequent urination	16	0.61 ± 1.15
Coughing and sneezing when urinating	13	0.72 ± 1.42
Reduced libido and desire	26	1.20 ± 1.42
Vaginal dryness during intercourse	26	0.83 ± 1.14
Avoid intimate relationship	6	0.38 ± 0.96
Tenderness or breast pain	3	0.27 ± 0.70
Bleeding or spotting	5	0.36 ± 0.91
Pain or leg cramps	19	0.97 ± 1.58

Similarly, regarding severity, hot flashes were found to be the most severe symptoms in our study, which is in agreement with the findings of a study on the people of shirazes in western Iran. Such variations are probably caused by the diversity of cultures, norms and traditions, and factors related to diet and lifestyle (26). Vaginal atrophy is a common symptom reported by menopausal women, which is associated with a group of symptoms such as itching, dryness, burning/soreness, irritation, discharge and painful intercourse (28, 29). In the same vein of research, vulvovaginal atrophy has been shown to be significantly related to female sexual dysfunction and complications of sexual desire, arousal, and orgasm around the world. It is expected that vulvovaginal atrophy, a physical condition caused by estrogen loss, exerts an adverse effect on the sexual desire of a woman and the vagina lubrication prior to the sexual intercourse. In most cases, the pain triggered by vaginal atrophy may prevent women from craving, initiating, or responding to sexual activity (4). 

The results of our study are inconsistent with the findings of Levine et al (4), Anderson et al (10) and Chedraui et al (8) which did not find any relationship between atrophy vaginal and sexual problem. Levine et al reported that women with female sexual dysfunction were 3.84 times more likely to develop vulvovaginal atrophy in comparison to women without female sexual dysfunction (95% CI: 2.99-4.94) (4).

Anderson et al revealed that vaginal discomfort was associated with two of the three domains of sexuality (10). Chedraui et al (8) showed that urogenital MRS scores predicted 59% of total FSFI score variance

However, we did not question participants about touching, kissing and hugging, which are usually used to compensate for the lack of penetrative intercourse. A study of GP practice in Sheffield showed that elderly couples had adapted by having more touching and hugging (14). In the literature, several subjective (e.g. vaginal dryness), and objective (e.g. pH, karyopycnotic index (KI) and maturation value (MI) - indicators have been adopted to measure the level of vaginal estrogenization (30). Future studies can assess the relationship between sexual dysfunction using both subjective and objective criteria.

Hot flashes, as the most common revolting symptom observed in approximately 70% of women (25), are often reported as intense feeling of warmth, sweating, flushing, and chills. Sweating is primarily reported in certain parts of the body like face, neck and chest (31). The results of our study were in line with the findings of Anderson et al (10) and McCoy et al (32). Anderson et al found that hot flashes were associated with two of the three domains of sexuality, and McCoy et al identified a close correlation between hot flashes, diminished estradiol levels, and reduced frequency of intercourse during the perimenopause. The results of our measurement invariance analyses were in conflict with the findings ofMerghati-Khoei (6), who reported no significant associations between middle-aged women’s sexual function severity of hot flashes, and hot flash frequency. 

Pujols also reported a relationship between sexual functioning, sexual satisfaction, and all variables related to the body image including sexual appeal, weight concern, and physical condition (11). Our finding suggest that certain aspects of body image such weight gain (p = 0.01) are associated with severe sexual problems. 

The results of our study suggest that psychological and physical symptoms such as anxiety and tenseness, memory loss, isolation, headache and neck pains, lack of stamina and energy, feeling bloated, lumbago, bleeding or spotting and leg pain or cramps are correlated with sexual problem in menopausal women as compared to women who lack such symptoms. It seem that there is a strong relationship between psychological, physical symptoms and sexual function; however, these findings should be treated cautiously as they are based on a cross sectional study. 

Another body of research has shown that female sexual function can be negatively affected by increased parity, which may alter vulvovaginal anatomy and sensitivity. Another line of studies have highlighted the role of pudendal nerve integrity and genital-sensory alterations with regard to female sexual dysfunction and old age (33) Our results seem to be consistent with the literature in terms of female parity, with parity >5 intensifying sexual problems in women compared to parity ≤ 5. The results, in line with previous research, reveal an inverse relation between age and sexual function (6, 34). Also, the results, in keeping with the literature, suggest that nonsmokers have higher FSFI scores than smokers (6).

## Conclusion

Women with a history of sexual problem experienced more intense menopausal symptoms. This study sheds more light on the link between sexual problems and menopausal symptoms, which can help healthcare professionals to offer a desirable package to their patients.

## References

[1] Saunamäki N, Andersson M, Engström M (2010). Discussing sexuality with patients: nurses' attitudes and beliefs. J Adv Nurs.

[2] Yencilek F, Attar R, Erol B, Narin R, Aydın H, Karateke A (2010). Factors affecting sexual function in premenopausal age women with type 2 diabetes: acomprehensive study. Fertil Steril.

[3] McAuliffe L, Bauer M, Nay R (2007). Barriers to the expression of sexuality in the older person: the role of the health professional. Int J Older People Nurs.

[4] Levine KB, Williams RE, Hartmann KE (2008). Vulvovaginal atrophy is strongly associated with female sexual dysfunction among sexually active postmenopausal women. Menopause.

[5] Berman JR, Berman LA, Werbin TJ, Goldstein I (1999). Female sexual dysfunction: anatomy, physiology, evaluation and treatment options. Curr Opin Urol.

[6] Merghati-Khoei E, Sheikhan F, Shamsalizadeh N, Haghani H, Yousofnia Pasha YR, Killeen T (2014). Menopause negatively impacts sexual lives of middle-aged Iranian women: a cross-sectional study. J Sex Marital Ther.

[7] Alizadeh Charandabi SM, Rezaei N, Hakimi S, Montazeri A, Khatami S, Karimi P (2012). Sexual function of postmenopausal women and its predictive factors: a community based study in Ilam, Iran, 2011. The Iranian Journal of Obstetrics, Gynecology and Infertility.

[8] Chedraui P, Pérez-López FR, Mezones-Holguin E, San Miguel G, Avila C, Collaborative Group for Research of the Climacteric in Latin America (REDLINC) (2011). Assessing predictors of sexual function in mid-aged sexually active women. Maturitas.

[9] Avis NE, Stellato R, Crawford S, Johannes C, Longcope C (2000). Is there an association between menopause status and sexual functioning?. Menopause.

[10] Greendale GA, Petersen L, Zibecchi L, Ganz PA (2001). Factors related to sexualfunction in postmenopausal women with a history of breast cancer. Menopause.

[11] Pujols Y, Seal BN, Meston CM (2010). The association between sexual satisfaction and body image in women. J Sex Med.

[12] Mishra G, Kuh D (2006). Sexual functioning throughout menopause: the perceptions ofwomen in a British cohort. Menopause.

[13] Hess R, Conroy MB, Ness R, Bryce CL, Dillon S, Chang CC (2009). Association of lifestyle and relationship factors with sexual functioning of women during midlife. J Sex Med.

[14] Parker S (2006). What barriers to sexual expression are experienced by older people in 24-hour care facilities?. Reviews in Clinical Gerontology.

[15] Ghazanfarpour M, Kaviani M, Abdolahian S, Bonakchi H, Najmabadi Khadijeh M, Naghavi M (2015). The relationship between women's attitude towards menopause and menopausal symptoms among postmenopausal women. Gynecol Endocrinol.

[16] Rosen R, Brown C, Heiman J, Leiblum S, Meston C, Shabsigh R (2000). The Female Sexual Function Index (FSFI): a multidimensionalself-report instrument for the assessment of female sexual function. J Sex Marital Ther.

[17] Sidi H, Abdullah N, Puteh SE, Midin M (2007). The Female Sexual Function Index(FSFI): validation of the Malay version. J Sex Med.

[18] Fakhri A, Pakpour AH, Burri A, Morshedi H, Zeidi IM (2012). The Female SexualFunction Index: translation and validation of an Iranian version. J Sex Med.

[19] Hilditch JR, Lewis J, Peter A, van Maris B, Ross A, Franssen E (2008). A menopause-specific quality of life questionnaire:development and psychometric properties. Maturitas.

[20] Whelan TJ, Goss PE, Ingle JN, Pater JL, Tu D, Pritchard K (2005). Assessment of quality of life in MA.17: a randomized, placebo-controlled trial of letrozole after 5 years of tamoxifen in postmenopausal women. J Clin Oncol.

[21] Yazdkhasti M, Keshavarz M, Khoei EM, Hosseini A, Esmaeilzadeh S, Pebdani MA, Et al (2012). The Effect of Support Group Method on Quality of Life in Post-menopausal Women. Iran J Public Health.

[22] Fallahzadeh H (2010). Quality of life after the menopause in Iran: a population study. Qual Life Res.

[23] Abedzadeh Kalarhoudi M, Taebi M, Sadat Z, Saberi F (2011). Assessment of quality of life in menopausal periods: a population study in kashan, iran. Iran Red Crescent Med J.

[24] Noroozian M (2012). The elderly population in iran: an ever growing concern in the health system. Iran J Psychiatry Behav Sci.

[25] Stearns V, Beebe KL, Iyengar M, Dube E (2003). Paroxetine controlled release in the treatment of menopausal hot flashes: a randomized controlled trial. JAMA.

[26] Ghazanfarpour M, Kaviani M, Abdolahian S, Bonakchi H, Najmabadi Khadijeh M, Naghavi M (2015). The relationship between women's attitude towards menopause and menopausal symptoms among postmenopausal women. Gynecol Endocrinol.

[27] Oldenhave A, Jaszmann LJ, Haspels AA, Everaerd WT (1993). Impact of climacteric on well-being. A survey based on 5213 women 39 to 60 years old. Am J Obstet Gynecol.

[28] Barlow DH, Cardozo LD, Francis RM, Griffin M, Hart DM, Stephens E (1997). Urogenital ageing and its effect on sexual health in older British women. Br J Obstet Gynaecol.

[29] Pastore LM, Carter RA, Hulka BS, Wells E (2004). Self-reported urogenital symptoms in postmenopausal women: Women's Health Initiative. Maturitas.

[30] Levis S, Griebeler ML (2010). The role of soy foods in the treatment of menopausalsymptoms. J Nutr.

[31] Freedman RR (2014). Menopausal hot flashes: mechanisms, endocrinology, treatment. J Steroid Biochem Mol Biol.

[32] McCoy N, Culter W, Davidson JM (1985). Relationships among sexual behavior, hot flashes, and hormone levels in perimenopausal women. Arch Sex Behav.

[33] Helpman L, Greenstein A, Hartoov J, Abramov L (2009). Genito-sensory analysis in women with arousal and orgasmic dysfunction. J Sex Med.

[34] Tomic D, Gallicchio L, Whiteman MK, Lewis LM, Langenberg P, Flaws JA (2006). Factors associated with determinants of sexual functioning in midlife women. Maturitas.

